# Regulated Expression of ADAMTS-12 in Human Trophoblastic Cells: A Role for ADAMTS-12 in Epithelial Cell Invasion?

**DOI:** 10.1371/journal.pone.0018473

**Published:** 2011-04-11

**Authors:** Alexander G. Beristain, Hua Zhu, Peter C. K. Leung

**Affiliations:** 1 Division of Cell Signaling Biology, Ontario Cancer Institute, Toronto, Ontario, Canada; 2 Department of Obstetrics and Gynecology, University of British Columbia, Vancouver, British Columbia, Canada; University of Birmingham, United Kingdom

## Abstract

Metastatic carcinoma cells exploit the same molecular machinery that allows human placental cytotrophoblasts to develop an invasive phenotype. As altered expression levels of ADAMTS (A Disintegrin And Metalloproteinase with ThromboSpondin repeats) subtypes have been associated with cancer progression, we have examined the function and regulation of members of this gene family in epithelial cell invasion using cultures of highly invasive extravillous cytotrophoblasts and the poorly invasive JEG-3 cytotrophoblast cell line as model systems. Of the multiple ADAMTS subtypes identified in first trimester human placenta and these two trophoblastic cell types, only ADAMTS-12 was preferentially expressed by extravillous cytotrophoblasts. Transforming growth factor-β1 and interleukin-1β, two cytokines that promote and restrain cytotrophoblast invasion *in vitro*, were also found to differentially regulate trophoblastic ADAMTS-12 mRNA levels. Loss- or gain-of-function studies confirmed that ADAMTS-12, independent of its proteolytic activity, plays a specific, non-redundant role in trophoblast invasion. Furthermore, we demonstrated that ADAMTS-12 regulated cell-extracellular matrix adhesion and invasion through a mechanism involving the αvβ3 integrin heterodimer. This study identifies a novel biological role for ADAMTS-12, and highlights the importance and complexity of its non-proteolytic domain(s) pertaining to its function.

## Introduction

Human placental development and function are dependent upon mononucleate cytotrophoblasts entering one of two distinct and mutually exclusive differentiation pathways [1], [2]. The villous pathway culminates in the formation of the syncytial trophoblast, a multinucleated, terminally differentiated cell that contributes to the majority of placental transport, immunoregulation and endocrine functions throughout pregnancy. Alternatively, cytotrophoblasts entering the extravillous pathway develop a highly invasive phenotype, which in turn allows these cells to invade deeply into the underlying maternal tissues and vasculature, thereby ensuring a continuous supply of blood to the developing fetus. Extravillous cytotrophoblast (EVT) invasion, unlike carcinoma cell metastasis, is a tightly controlled, developmental process [3], [4].

The onset of trophoblastic cell differentiation along the extravillous pathway is dependent upon the proteolytic degradation and/or activation of distinct extracellular matrix (ECM) components, and regulated changes in cell-cell and cell-ECM interactions [5],[6]. Consequently, most studies to date have focused upon the roles of matrix metalloproteinases/tissue inhibitors of metalloproteinases (MMPs/TIMPs), cytokines, integrins, and cadherins in this cellular event. However, there is increasing evidence to suggest that the regulated expression of members of the ADAMTS (A Disintegrin And Metalloproteinase with ThromboSpondin repeats) gene family may represent a significant molecular mechanism for mediation of the terminal differentiation of human cytotrophoblasts and the development of an invasive phenotype.

The ADAMTS are a family of secreted proteins that are generally characterized by four structural and functional domains; an amino terminal prodomain, a catalytic domain, a disintegrin-like domain, and an ECM-binding domain (composed of a central thrombospondin type 1 (TSP1) motif, a spacer region and a variable number of TSP-like repeats) at the carboxyl terminal of the mature protein species [7], [8]. Thus, all members of this gene family have the potential to act as metalloproteinases and to regulate cell adhesion. Some ADAMTS subtypes have been further subclassified according to the presence of additional C-terminal modules or the identification of common substrates. Furthermore, distinct ADAMTS subtypes have also been shown to play integral roles in the growth and development of tissues and in the onset and progression of degradative diseases including cancer [9]–[11], arthritis [12], Alzheimer's disease and a number of inflammatory and thrombotic conditions [13], [14].

Here, we have examined the function and regulation of the ADAMTS in the differentiation of human cytotrophoblasts along the extravillous pathway. We first determined that multiple ADAMTS subtypes are present in first trimester human placenta, in cultures of invasive EVTs propagated from these tissues and in the poorly invasive JEG-3 choriocarcinoma cell line. Of these, only ADAMTS-12 was found to be present in EVTs at significantly higher levels than in JEG-3 cells. The ability of transforming growth factor (TGF)-β1 and interleukin (IL)-1β, two cytokines assigned counter-regulatory roles in human placentation [3], [15], to differentially regulate ADAMTS-12 mRNA levels in cultures of EVTs supported our hypothesis that this ADAMTS subtype confers an invasive phenotype on human trophoblastic cells. Loss- or gain-of-function studies subsequently confirmed that this ADAMTS subtype, independent of its intrinsic proteolytic activity, plays an active and dominant role in human trophoblastic cell invasion *in vitro* through a mechanism controlling cell-ECM interaction. Specifically, ADAMTS-12 modulated cell invasion by regulating the expression and function of the αvβ3 integrin heterodimer.

## Materials and Methods

### Tissues

Samples of first trimester placental tissues were obtained from women undergoing elective termination of pregnancy (gestational ages ranging from 6–12 weeks). The use of these tissues was approved by the Committee for Ethical Review of Research on the use of human subjects, University of British Columbia. All women provided informed written consent.

### Tissue culture

EVT cultures were propagated from first trimester placental explants essentially as described [16]. The purity of the EVT cultures was determined by immunostaining for human cytokeratin filaments 8 and 18. Only cultures that exhibited 100% immunostaining for these cytokeratins were included. 5×10^6^ EVTs were grown to 80% confluency and treated with TGF-β1 (0.1–10 ng/ml) or IL-1β (1–1000 IU/ml) for 24 h, or TGF-β1 (5 ng/ml) or IL-1β (100 IU/ml) for 0–48 h. EVTs cultured in vehicle (ethanol) served as controls. Specific cultures were subjected to a function-perturbing monoclonal antibody against human TGF-β1 (Sigma Aldrich; 10 µg/ml; clone 9016.2) or IL-1β (Sigma Aldrich;100 IU/ml; clone 8516.311) for 24 h. JEG-3 trophoblastic cell were purchased from ATCC, Manassas, VA, USA. On-going cultures were maintained in DMEM containing 25 mM glucose, L-glutamine, antibiotics (100 U/ml penicillin, 100 µg/ml streptomycin) and supplemented with 10% FBS.

### Primer Design and preparation of cDNA Probes

Primer sets for ADAMTS-1 through -12 [17] or GAPDH were synthesized at the NAPS Unit, UBC. The nucleotide sequences of primers, optimized PCR conditions, and the sizes of the PCR products are listed in Table S1. To generate cDNA probes for each ADAMTS or GAPDH, PCR products were generated from human placental tissue, subcloned into the PCR II vector and confirmed by nucleotide sequencing. A second set of ADAMTS-12-specific primers, in which a stretch of nucleotides corresponding to a sequence present within the target ADAMTS-12 PCR product was incorporated into the 3′-end of the forward primer; These were used for quantitative competitive (QC)-PCR analysis of ADAMTS-12 mRNA levels in cultures treated with TGF-β1 and IL-1β. This follows a similar approach as reported for examining urokinase plasminogen activator/plasminogen activator inhibitor-1 and MMP/TIMP mRNA levels [18].

### Semiquantitative PCR and Southern blot analysis

Total RNA was prepared from tissue samples or cells using an RNeasy Mini Kit (Qiagen, Inc, CA) following the manufacturer instructions. Aliquots (∼1 µg) of the total RNA extracts were then reverse-transcribed into cDNA using a First Strand cDNA Synthesis Kit (Amersham Pharmacia Biotech, Oakville, ON, Canada). Semiquantitative PCR was performed using the primer sets listed in Table S1. All PCR reactions were performed on 3 separate occasions (n = 3). PCR products were separated by standard electrophoresis followed by Southern blotting according to the methods of MacCalman *et al* [19]. The autoradiograms were scanned using a laser densitometer (Scion Corporation, Frederick, MD, USA) and the absorbance values of the distinct ADAMTS PCR products normalized relative to the corresponding GAPDH value.

### QC-PCR

The QC-PCR strategy employed in these studies is based upon the competitive co-amplification of a known amount of competitive ADAMTS-12 PCR product added to aliquots of first strand cDNA prepared from our primary cultures of EVTs. The PCR conditions were: 1 min at 94°C, 1 min at 58.5°C and 1.5 mins at 72°C for 28 cycles followed by a final extension at 72°C for 15 min. The resultant target and competitive ADAMTS-12 PCR products were separated using gel electrophoresis. PCR products identity was confirmed by subcloning and DNA sequencing (data not shown). To determine the optimal amount of competitive cDNA to be added to each reaction, PCR was performed using either a fixed amount of template cDNA combined with decreasing concentrations of competitive cDNA or conversely, a fixed concentration of competitive cDNA combined with decreasing amounts of template cDNA. The intensity of ethidium bromide staining of the PCR products was analysed by UV densitometry, and volume counts determined using the Scion Image computer software. The competitive ADAMTS-12 cDNA was subsequently added to each reaction at a concentration of 4.88×10^−3^ pg/µl.

### Cell lysis, immunoprecipitation and immunoblot analysis

Cultures of JEG-3 cells or EVTs were washed in PBS and incubated in cell extraction buffer (Biosource International, Camarillo, CA) supplemented with 1.0 mM PMSF and Complete Mini proteinase-inhibitor cocktail (Roche) for 30 minutes. Protein concentrations were determined using a BCA kit (Pierce Chemicals, Rockford, IL, USA). Lysates (500 µg) were immunoprecipitated with an αv integrin antibody (Chemicon International) overnight at 4°C followed by incubation with protein G plus protein A agarose beads (Invitrogen) for 1 hr. The precipitated protein complexes were washed at 4°C in RIPA buffer without sodium deoxycholate or SDS. For immunoblotting, 30 µg of cell protein lysate was resolved by SDS-PAGE and transferred to nitrocellulose membranes. The membranes were probed using polyclonal antibodies against the carboxyl terminal of human ADAMTS-12 (Santa Cruz Inc, Santa Cruz, CA, USA) and ADAMTS-1 (AbCAM), and monoclonal antibodies directed against αv, β1, β3 integrin subunits (Chemicon International), phospho-397 FAK and FAK (Cell Signaling). The blots were reprobed with a monoclonal antibody directed against human β-actin (Sigma Aldrich, St, Louis, MO) to assess protein loading.

### siRNA transfection

siRNA (Xeragon Inc, Germantown, MD; 13.5 µg/100 mm^2^ culture dish) targeting the human ADAMTS-12 mRNA transcript (5′-AAGCCCGTCCCTCCACCTACA-3′) was transfected into EVTs using TransMessenger transfection reagent (Qiagen) according to manufacturer's protocol. EVTs transfected with a non-silencing, scrambled siRNA (5′- ATTTCTCCGAACGTGTCACGT-3′) or cultured in the presence of transfection reagent alone, served as negative controls. The concentration of siRNAs used was selected based on previous studies of primary EVT cultures [20]. Following optimization of the TransMessenger∶siRNA concentration ratio, all experiments were performed for at least 24 h.

### Expression Vectors

Mammalian expression vectors (pcDNA3.1; Invitrogen, Carlsbad, CA) containing either a full length human ADAMTS-12 cDNA (pcDNA3-ADAM-TS12-HA) or a full-length human ADAMTS-12 cDNA in which the catalytic domain had been inactivated by site directed mutagenesis (pcDNA3-ADAM-TS12-MUT) were generously provided by Dr. S. Cal (Universidad de Oviedo, Spain) herein abbreviated as A12FL and A12Mut, respectively. These constructs have been described [21]. A12Mut cDNA construct has two point mutations in the metalloproteinase domain, where the thymine (465) and adenine (466) have been substituted for adenine and cytosine resulting in the translated protein consisting of a glucine amino acid instead of histidine thus rendering the protein catalytically inactive. A full-length human ADAMTS-1 cDNA (Genbank Accession No. NM006988) was purchased from Origene (Rockville, MD, USA) and cloned into pcDNA3.1. A clone (A1) containing the ADAMTS-1 cDNA in the forward orientation was subsequently identified by DNA sequencing. A pcDNA3.1 expression vector containing the β-galactosidase gene (pcDNA3-LacZ; Invitrogen) was used to determine transfection efficiency and served as a control for these studies.

### Generation of stably transfected JEG-3 cell lines

Stable transfections were performed to establish clonal JEG-3 cell lines constitutively expressing A12FL, A12Mut, A1 or LacZ. Each expression vector (1.0 µg/ml) was transfected using Exgen 500 transfection reagent (Fermentas, Burlington, ON, Canada) according to the manufacturer's protocol. Selection began 48 h post transfection using 400 µg/ml G418 in DMEM, and subcolonies were isolated by limiting dilution and expanded into cell lines maintained in the selection medium. At least three independent clones were selected per construct and protein expression confirmed by Western blotting (data not shown).

### Transwell invasion assays

Cell invasion assays were performed using Transwells fitted with Millipore Corp. membranes coated with a thin layer of growth factor-reduced Matrigel (6.5-mm filters, 8-µm pore size) as described [22]. Briefly, 2×10^4^ cells/200 µl of DMEM supplemented with 1% FBS were plated in the upper chambers and cultured for 24 h (EVTs) or 48 h (JEG-3 cells). Cells from the upper surface of the Matrigel layer were removed by gentle swabbing, while transmigrated cells attached to the membrane were fixed in 4% paraformaldehyde and stained with eosin. The filters were rinsed with water, excised from the Transwells, and mounted upside-down onto glass slides. Invasion indices were determined by counting the number of stained cells in 10 randomly selected, non-overlapping fields at 400× magnification using a light microscope. Cell invasion was tested in triplicate wells, on three independent occasions. A subset of these experiments were performed in the presence of a synthetic RGD (Arg-Gly-Asp) or control peptide (1 mM; BIOMOL International, Plymouth Meeting, PA) following procedures of Buckley *et al* [23], a bicyclic RGD peptide (H-Glu[cyclo(Arg-Gly-Asp-D-Phe-Lys)]_2_; Peptides International) shown to possess high affinity towards αvβ3 integrin (IC_50_ = 0.9 nM) with low affinity for αvβ5 and α_IIB_β3 integrins (IC_50_ = 10 nM) and a cyclo (Arg-Ala-Asp-D-Phe-cys; Peptides International) peptide used as a negative control. In addition to the RGD peptides, a monoclonal function perturbing αv integrin antibody (Chemicon International; mouse anti-integrin αv antibody; clone AV1) was used in Transwell invasion experiments [24]. Prior to seeding cells into invasion chambers, 5.4×10^5^ cells in media suspension were pre-incubated in the presence of the indicated peptide/antibody for 30 min.

### Cell aggregation assays

Cell aggregation assays were performed using the cell hanging-drop method [25]. Briefly, trypsinized single cell suspensions (1.5×10^5^ cells/ml) treated with EDTA and passaged through 20 µm nylon sieves were prepared in DMEM media containing 10% FBS. From these suspensions, three 20 µl droplets were pippetted onto the underside of 6 cm culture dish lids, which were carefully inverted and placed onto its bottom well containing 2 ml DMEM media and cultured for 2 or 8 h. After incubation, culture dish lids were again inverted and glass coverslips were mounted onto the cell-drop suspensions. The extent of cellular aggregation was quantified by counting cell aggregates under a light microscope fitted with a digital camera (200× magnification); aggregates were allocated into 3 groups: <5 cells/aggregate, 5–15 cells/aggregate, and >15 cells/aggregate. Each experiment was repeated a minimum of three times.

### Native ECM-binding assay

Stably transfected JEG-3 cells were plated in 24-wells in triplicate at high density and allowed to grow to near confluency for 24 h. Cell media was replaced with fresh DMEM media and cultured for an additional 48 h to allow for matrix deposition. Cell monolayers were removed by incubation with 20 mM NH_4_OH (2×5 min) according to Gospodarowicz *et al* [26] followed by washing in 1× PBS. JEG-3 cells growing in parallel were trypsinized, passaged through a 40 µm nylon sieve, plated onto the ECM deposited by the transfected JEG-3 cell lines and cultured for 30 min. Cells were washed vigorously in 1×PBS, fixed in 4% paraformaldehyde and stained with eosin. ECM binding ability was determined by counting the number of cells that had adhered to the deposited ECM under a microscope.

### ECM Cell Adhesion Assay

A fluorimetric ECM cell adhesion array kit (Chemicon International) allowed us to screen for and quantitate the binding affinity of cells to 7 different human ECM proteins. Collagen I, collagen II, collagen IV, fibronectin, laminin, tenascin, and vitronectin were pre-coated onto 96-well microtiter plates arranged in 12×8-well strips. In each 8-well strip, one BSA-coated well served as a negative control. Following the manufacturers instructions, we seeded 1×10^5^ cells suspended in DMEM (100 µl) onto the coated ECM-substrates and analysed for the cells ability to bind to the respective ECM proteins using a fluorescence plate reader (485/530 nm). The plating of trophoblastic cells onto 8-well ECM protein coated strips was performed on three independent occasions (n = 3).

### Alpha and Beta Integrin Binding Assay

A fluorimetric α/β Integrin-Mediated Cell Adhesion Array Combo Kit (Chemicon International) was used to assess the integrin repertoire expressed on the cell surface. The kit is comprised of two 96-well plates: an α-integrin binding 96-well plate and a β-integrin 96-well plate. Each kit uses mouse monoclonal antibodies generated against human α (α1, α2, α3, α4, α5, αv, αvβ3), and β (β1, β2, β3, β4, β6, αvβ5, α5β1) integrins/subunits, that are immobilized onto goat-anti-mouse antibody coated microtiter plates. Goat anti-mouse antibody coated wells served as negative assay controls. Following the manufacturers protocol, 1×10^5^ cells suspended in DMEM (100 µl) were plated into the 96-wells and incubated for 2 h. Unbound cells were washed away, and adherent cells were lysed and integrin binding function/expression was detected using a fluorescent DNA-binding dye provided with the integrin binding kit using a fluorescence plate reader (485/530 nm). Integrin binding assays were performed on three independent occasions (n = 3).

### Statistical Analysis

Statistical differences between the absorbance values were assessed by the analysis of variance (ANOVA) using GraphPad Prism 4 computer software (San Diego, CA, USA), and significant differences between the means were determined using Dunnett's test [16]. Cellular invasion indices were analyzed by one-way ANOVA followed by the Tukey multiple comparison test [22]. The differences were accepted as significant at P<0.05.

## Results

### Characterization of the ADAMTS subtypes present in human placental tissue

**Figure 1:**
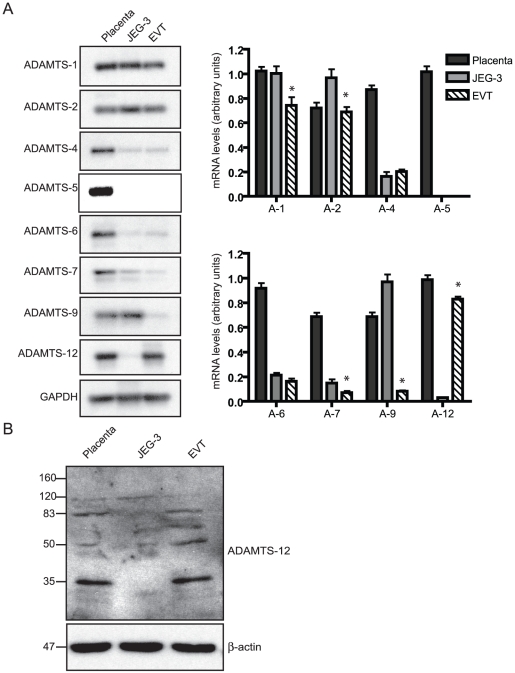
Characterization of the ADAMTS subtypes present in human placenta and trophoblastic cells. (A) Representative autoradiograms of Southern blots containing PCR products synthesized from total RNA from first trimester placenta, JEG-3 cells or EVTs using primers specific for the indicated ADAMTS or GAPDH. The presented results are densitometry readings showing mean ± SEM (n≥4; * = P≤0.05). (B) A representative Western blot containing total protein prepared from first trimester placenta, EVTs or JEG-3 cells, was probed with a polyclonal antibody against ADAMTS-12. The blots were re-probed with monoclonal against human b-actin. The molecular weight markers (kDa) are shown to the left.

ADAMTS-1, -2, -4, -5 (also known as ADAMTS-11), -6, -7, -9, and -12 mRNA levels were detected in first trimester human placenta (Figure 1A). In contrast, ADAMTS-3, -8 and -10 mRNA were not detected in these placental tissues but were readily detectable in our positive controls (data not shown). This repertoire of ADAMTS subtypes was maintained in both highly invasive EVTs and poorly invasive JEG-3 cells, with the exception of ADAMTS-5, which was not detected in either trophoblastic cell type examined in these studies (Figure 1A). However, ADAMTS-12 mRNA levels in EVTs were significantly higher than those detected in JEG-3 cells. In contrast, ADAMTS-1, -2, -7, and -9 mRNA levels were significantly lower in EVTs, whereas there was no significant difference between the levels of the ADAMTS-4 and -6 mRNA transcripts present in these two trophoblastic cell types.

### High ADAMTS-12 expression is observed in invasive human trophoblastic cells

As previous work has failed to detect ADAMTS-12 in normal human tissues and cells [21], we performed Western blot analysis to confirm that this ADAMTS subtype was expressed in first trimester human placenta *in vivo* and in trophoblastic cells *in vitro*. Major ADAMTS-12 protein species (83, 50 and 35 kDa), corresponding to a C-terminal fragments generated by post-translational cleavage of the ADAMTS-12 zymogen [21] were detected in the total protein lysates prepared from these placental tissues or cultures of EVTs or JEG-3 cells (Figure 1B). Furthermore, and in agreement with our PCR data, ADAMTS-12 protein expression levels were found to be significantly higher in EVTs as compared to JEG-3 cells.

### Cytokines regulate ADAMTS-12 mRNA levels in EVTs

**Figure 2:**
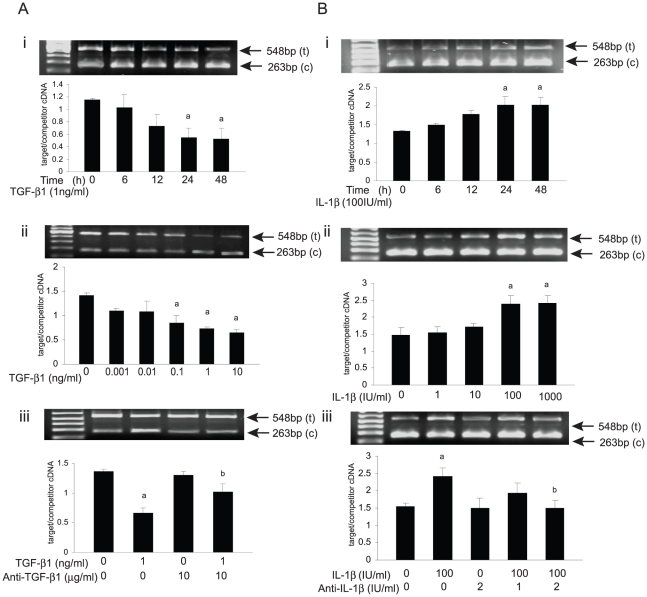
Regulatory effects of TGF-β1 and IL-1β on ADAMTS-12 mRNA expression levels in EVTs. (A) QC-PCR analysis of ADAMTS-12 mRNA levels in EVTs cultured in the presence of (i) TGF-β1 for 0–48 h, (ii) 0–10 ng/ml of TGF-β1 for 24 h, (iii) in the presence of vehicle, TGF-β1 with or without an antibody against TGF-β1 for 24 h. (B) Similar analyses following (i) 0–48 IL-1β, (ii) 0–1000 IU/ml of IL-1β for 24 h, (iii) in the presence of vehicle, IL-1β with or without an antibody directed against IL-1β for 24 h. Representative photomicrographs of the resultant ethidium bromide-stained gels are presented (t and c denote the target and competitive PCR transcripts respectively). The data are presented mean ± SEM (n = 3; a = P≤0.05 vs. untreated control; b = P≤0.05 vs. cytokine alone).

TGF-β1 and IL-1β are spatiotemporally expressed at the maternal-fetal interface and have been shown to be potent regulators of human trophoblastic cell invasion *in vitro* [3], [15]. In view of these observations, we examined the ability of these two cytokines to regulate ADAMTS-12 mRNA levels in primary cultures of EVTs in a time- and dose-dependent manner. ADAMTS-12 mRNA was detected in all of the EVT cultures (Figures 2A and 2B). The addition of vehicle (ethanol) to the culture medium of EVTs, which served as a negative control, had no significant effect on ADAMTS-12 mRNA levels in these cells at any of the time points examined (data not shown).

A significant decrease in ADAMTS-12 mRNA levels was detected in EVTs cultured in the presence of TGF-β1 (1 ng/ml) for 24 h with the levels of this mRNA transcript continuing to decrease until the termination of these studies at 48 h (Figure 2A). The addition of increasing concentrations of TGF-β1 to the culture medium of these cells demonstrated that ADAMTS-12 mRNA levels in EVTs were regulated in a dose-dependent manner (Figure 2A). A function-perturbing monoclonal antibody directed against TGF-β1 abolished the TGF-β1-mediated decrease in ADAMTS-12 mRNA levels in these primary cell cultures (Figure 2A). In contrast, IL-1β (100 IU) caused a continuous and significant increase in ADAMTS-12 mRNA levels in EVTs over time in culture (Figure 2B). Maximum levels of this mRNA transcript were subsequently detected in EVTs cultured in the presence of the highest concentration (1000 IU) of this cytokine examined in these studies (Figure 2B). A function-perturbing monoclonal antibody directed against IL-1β was also found to attenuate the increase in ADAMTS-12 mRNA levels observed in EVTs cultured in the presence of this cytokine (Figure 2B).

Our initial findings demonstrated that similar to many other normal and malignant epithelial cells examined to date [21], ADAMTS-12 expression levels are higher in human trophoblastic cells with an invasive phenotype. The ability of cytokines to differentially regulate ADAMTS-12 mRNA levels in primary cultures of EVTs provided further evidence that ADAMTS-12 contributes to the highly regulated invasion of human trophoblastic cells. In view of these observations, we hypothesized that altered expression levels of ADAMTS-12 would, in turn, modulate the invasive capacity of these cells.

### Silencing ADAMTS-12 expression reduces the invasive capacity of EVTs

**Figure 3:**
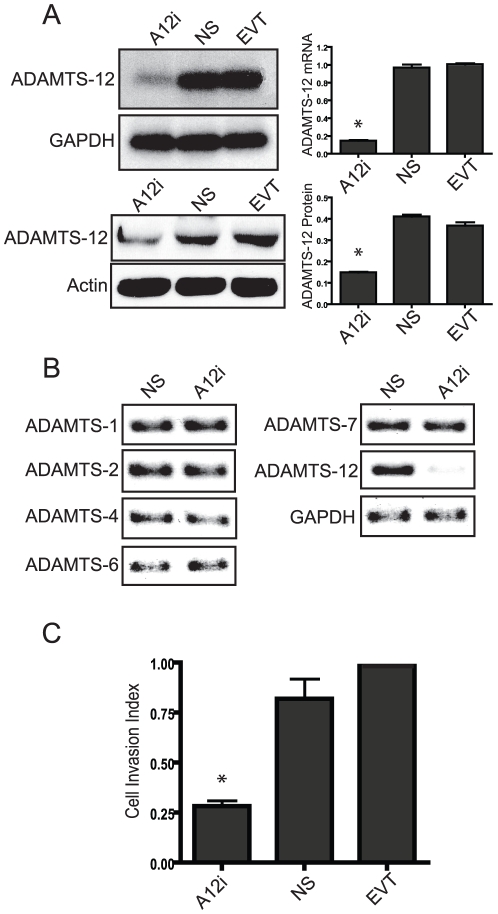
Reduced ADAMTS-12 expression levels decrease EVT invasion. (A) EVTs were transfected with siRNA specific for ADAMTS-12 (A12i), a scrambled control siRNA (NS) or cultured in the presence of transfection reagent alone (EVT) for 48 h. Cultures were harvested for total RNA or protein. The results are presented (mean ± SEM; n≥4) in the bar graphs (* = P≤0.05). Representative Western blots prepared using total protein extracts and probed with antibodies directed against either ADAMTS-12 or b-actin are shown. The results are presented (mean ± SEM; n≥4) in the bar graphs (* = P≤0.05). (B) Photomicrographs of ethidium bromide stained agarose gels containing PCR products generated using primers specific for ADAMTS-1, -2, -4, -5, -6, -7 -12 or GAPDH. (C) EVTs were subjected to Matrigel invasion anlaysis as described in Methods. EVTs cultured in the presence of transfection reagent alone (EVT) were given an arbitrary invasion index = 1.0. Each cell line was plated in triplicate wells with the experiment repeated on at least 3 independent occasions (n = 3). The results are presented as mean ± S.E.M. in the bar graph (* = P≤0.05, compared to EVT control).

In order to decrease ADAMTS-12 expression in primary cultures of EVTs, we obtained 4 distinct siRNA sequences complementary to the human ADAMTS-12 mRNA transcript. Transient transfection with these siRNAs demonstrated that only one siRNA (herein referred to as A12i) significantly decreased ADAMTS-12 mRNA and protein expression levels in these cell cultures after 24 h (Figure 3A). In contrast, there was no significant difference between ADAMTS-12 mRNA and protein levels in the two control cultures; EVTs transfected with a non-silencing (NS), scrambled siRNA or cultured in the presence of transfection reagent alone (EVT; Figure 3A). To verify the knockdown specificity of A12i and to determine whether there were compensatory changes in the levels of other ADAMTS subtypes, PCR was performed using template cDNA generated from EVTs transfected with either the A12i or control siRNA and the primers specific for each ADAMTS subtype previously identified in these cells. Additionally, A12i did not affect the levels of ADAMTS-1, -2, -4, -6, and -7 mRNA transcripts (Figure 3B). These data confirm specificity of the siRNA used to knock-down endogenous ADAMTS-12 mRNA levels in EVT cultures, and also demonstrate that a reduction in ADAMTS-12 expression is not accompanied by compensatory changes in the expression of other ADAMTS subtypes.

We next tested whether a reduction in ADAMTS-12 expression levels in EVTs would result in a concomitant decrease in their invasive capacity. Matrigel-coated Transwell invasion chamber assays revealed that the number of cells that penetrated the Matrigel barrier and adhered to the underside of the Millipore filters were found to be significantly and consistently fewer in cultures of EVTs transfected with A12i compared to controls (Figure 3C).

### Exogenous ADAMTS-12 induces invasion in JEG-3 cells

**Figure 4:**
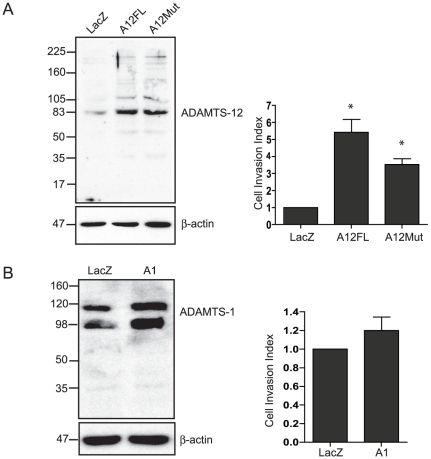
Exogenous ADAMTS-12 expression specifically increases the invasive capacity of JEG-3 cells, independent of its intrinsic proteolytic activity. (A) A representative Western blot depicting ADAMTS-12 protein levels in JEG-3 cells stably transfected with LacZ, A12FL or A12Mut. (B) A Western blot depicting ADAMTS-1 protein levels in JEG-3 cells stably transfected with A1 or LacZ. Expression levels of each ADAMTS were standardized to b-actin. Invasion assays were also performed using these cell lines and are represented as histograms (A and B). The results are presented as mean ± S.E.M; n≥10 in the bar graphs (* = P≤0.05, where * denotes a significantly different value compared to LacZ).

To determine whether exogenous ADAMTS-12 expression could confer an invasive phenotype on trophoblastic cells, JEG-3 cells were stably transfected with the expression vector A12FL; the catalytic activity of the expressed ADAMTS-12 protein species has been verified in previous studies derived from the same expression construct used in this study [21], [27]. Similar to our preceding observations, a major ADAMTS-12 protein species (83 kDa) was readily detectable in these JEG-3 cells but not in those transfected with the control LacZ expression vector (Figure 4A). In addition to the major protein species detected, exogenous expression of ADAMTS-12 also revealed a minor protein species of approximately 110 kDa and 50 kDa. Utilizing our transwell invasion system, we next determined that the invasive capacity of JEG-3 cells exogenously expressing ADAMTS-12 was significantly and consistently higher than the control cultures (Figure 4A).

### Proteinase-dead ADAMTS-12 increases invasion of JEG-3 cells

As a first step in characterizing the molecular mechanisms underlying ADAMTS-12-mediated cellular invasion, JEG-3 cells were stably transfected with the mammalian expression vector containing the proteinase-dead form of ADAMTS-12 (A12Mut) [21]. Similar to our preceding findings, a major ADAMTS-12 protein species (83 kDa) was readily detectable in these JEG-3 cells following transfection with this vector (Figure 4A). Furthermore, Matrigel invasion analysis demonstrated that there was no significant difference in the invasive capacity of these cells and those expressing A12FL(3.5±0.9 (A12Mut) vs. 5.43±1.3 (A12FL); p≤0.18), with both cell lines being consistently and significantly more invasive than the control cultures of JEG-3 cells (Figure 4A).

### ADAMTS-1 cannot mimic the effects of ADAMTS-12 induced invasion on JEG-3 cells

To determine whether an increase in the expression levels of another ADAMTS subtype, other than ADAMTS-12, could increase the invasive capacity of human trophoblastic cells *in vitro*, JEG-3 cells were stably transfected with the mammalian expression vector, pcDNA3-ADAM-TS-1 (A1). Western blot analysis revealed the presence of two distinct ADAMTS-1 protein species in these JEG-3 cell cultures and, albeit at lower levels, in those transfected with the control LacZ vector. These 2 isoforms of ADAMTS-1 correspond to the zymogen and mature active form [28]. However, higher ADAMTS-1 expression levels in JEG-3 cells did not result in a concomitant increase in their invasive capacity (p≤0.31; Figure 4B).

### Exogenous ADAMTS-12 expression does not affect cellular aggregation

Cell motility is regulated in part by the differential expression and function of cell adhesion molecules expressed on the cell surface [29]. To investigate whether exogenous ADAMTS-12 in JEG-3 cells could affect cell-cell binding affinity, A12FL, A12Mut, and LacZ stably transfected JEG-3 cells were subjected to a cell hanging-drop assay. Figure S1 quantitatively shows that neither full-length nor proteinase-dead ADAMTS-12 alters the aggregative capacity of JEG-3 trophoblastic cells assayed at 2 and 8 h timepoints, suggesting that cell-cell interactions are not modulated by exogenous ADAMTS-12 expression.

### ADAMTS-12 affects cell-ECM affinity in JEG-3 cells

**Figure 5:**
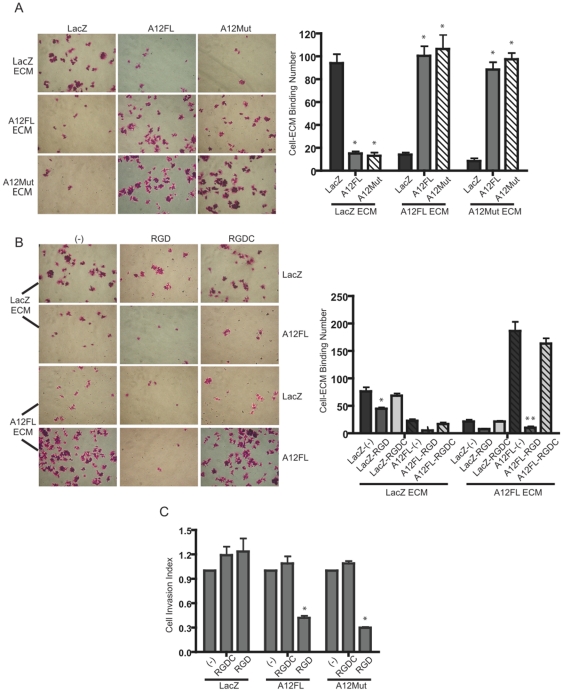
ADAMTS-12 regulates cell-extracellular matrix binding and invasion through an RGD-dependent mechanism. (A) Panels of photomicrographs of JEG-3 cells stably transfected with A12FL, A12Mut or LacZ. Cells were seeded onto 24-well plates pre-coated with ECM deposited by the same JEG-3 cell lines in a combinatorial fashion, and cultured for 30 min. Adjacent histograms show the number of cells that had bound to the pre-deposited native ECM, as described in Methods. (B) JEG-3 cells stably transfected with A12FL or LacZ were subjected to the native cell-ECM binding experiment described above, however cells were treated with either an RGD inhibiting peptide (RGD; 1 mM), a control peptide (RGDC; 1 mM) or untreated DMEM media (-). (C) A histogram representing the invasive indices of JEG-3 cells stably transfected with LacZ, A12FL or A12Mut. Cells were cultured in Matrigel-coated Transwell invasion chambers in complete DMEM media containing the RGD-inhibiting peptide (RGD; 1 mM), control peptide (RGDC; 1 mM) or complete DMEM media without treatment (-). Untreated cell lines were given an arbitrary invasion index of 1, and the invasive capacity of cells cultured in the presence of the control or RGD peptide were determined by generating an invasion index corresponding to their control cell line. All assays were performed in triplicate and performed on three independent occasions (n = 3; * = P≤0.05, compared to controls).

To further elucidate the potential molecular mechanism by which ADAMTS-12 elicits an invasive phenotype in poorly-invasive trophoblastic cells, we analyzed whether cell-ECM binding affinities differed between JEG-3 cells exogenously expressing full-length ADAMTS-12, proteinase-dead ADAMTS-12 or LacZ. To do this, we cultured A12FL, A12Mut, and LacZ transfected JEG-3 cells in a combinatorial fashion on extracellular matrices deposited by these same JEG-3 transfectants. JEG-3 cells exogenously expressing ADAMTS-12 significantly adhered more readily to their own native ECM (Figure 5A). Likewise, JEG-3 cells exogenously expressing proteinase-dead ADAMTS-12 or LacZ bound with high affinity to their own ECMs. JEG-3 cells expressing ADAMTS-12 adhered to ECM deposited by proteinase-dead ADAMTS-12 expressing cells and vice versa with high affinity, whereas control LacZ-expressing cells bound with less affinity to ECM deposited by A12FL or A12Mut expressing cells. The ability of the full-length and proteinase-dead ADAMTS-12 expressing JEG-3 cells to bind to ECM deposited by the control LacZ expressing cells were also significantly ablated. A possible explanation for the observed differences in cell-ECM binding could lie in the ability of these cells to differentially synthesize and deposit specific ECM proteins. To investigate this possibility, we analyzed by semi-quantitative PCR analysis the mRNA levels of vitronectin, laminin, tenascin and fibronectin, ECM proteins known to be expressed in the human endometrium [30]–[32] and therefore would be encountered by invading EVTs (Figure S2). While we did not observe changes in expression levels of these mRNA transcripts in A12FL and LacZ JEG-3 cells, we did note strong differences in vitronectin, laminin and tenascin mRNA levels between primary cultures of EVTs and JEG-3 cells. Specifically, laminin was expressed in greater levels in JEG-3 cells than in EVTs, while vitronectin and tenascin were detected exclusively in EVTs. Taken together, these data suggest that ADAMTS-12 facilitates cell-matrix interactions by regulating cell-ECM adhesion through a mechanism(s) independent of its endogenous proteolytic activity.

### RGD peptide disrupts ADAMTS-12 mediated cell-ECM binding and invasion

In an attempt to elucidate the mechanism by which ADAMTS-12 regulates cell-ECM binding, we performed native ECM-binding assays as described above for JEG-3 cells stably transfected with ADAMTS-12 or LacZ in the presence of a synthetic RGD inhibiting peptide, control peptide, or culture media alone. RGD peptide treatment significantly abrogated cell-ECM binding in all ADAMTS-12 and LacZ transfected cell populations, whereas ECM binding affinities of cells cultured in untreated media or with the control peptide were not affected (Figure 5B). Interestingly, the extent of RGD peptide-mediated cell-ECM binding inhibition was significantly more pronounced in ADAMTS-12 expressing cells than in LacZ expressing cells cultured on their own native ECM (p≤0.01 versus p≤0.05). Similar to our previous findings, exogenous ADAMTS-12 altered cell-binding affinities to native ECM deposited by JEG-3 cells transfected with A12FL or LacZ, though in this experiment it was shown that ADAMTS-12-mediated cell-ECM adhesion is in part regulated through an RGD-dependent mechanism. This finding sheds new light on the putative mechanism by which ADAMTS-12 modulates cell-ECM interaction, namely through an RGD sequence molecular event most likely involving members of the integrin cell adhesion molecule family.

Cell invasion is regulated in part by cell-ECM interactions mediated primarily through integrin receptors expressed on the cell surface [33]. Our findings demonstrating that ADAMTS-12 could modulate JEG-3 cell-ECM adhesion in an RGD dependent manner suggested that ADAMTS-12-mediated cell invasion could also be regulated through a similar RGD/integrin-dependent mechanism. To test this hypothesis, we cultured A12FL, A12Mut, and LacZ expressing JEG-3 cells in Matrigel-coated Transwell invasion chambers in the presence of the RGD inhibiting peptide. Cells were also cultured in the presence of a control peptide that served as a technical control, or were cultured in complete DMEM media. There were no significant differences in the invasive indices of LacZ transfected JEG-3 cells when cultured in the presence of either the RGD or control peptides (Figure 5C). However, the invasive indices of JEG-3 cells stably transfected with A12FL or A12Mut cultured in the presence of the RGD peptide were significantly less than the invasive indices of the same cells cultured in presence of the control peptide or media alone (Figure 5C).

### ADAMTS-12 promotes an invasive EVT-like cell-ECM binding phenotype

**Figure 6:**
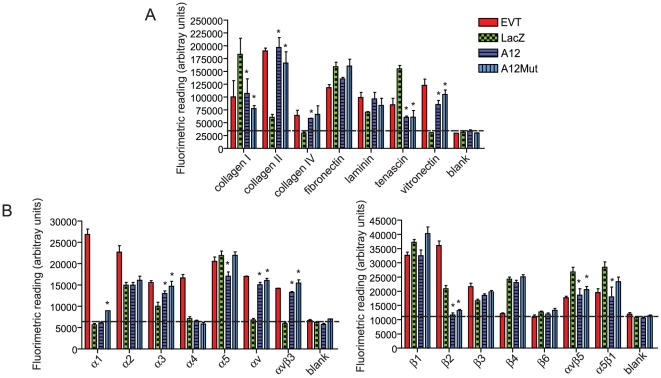
ADAMTS-12 modulates ECM binding mimicking that of an invasive phenotype. (A) Histogram depicting binding affinities of EVTs and JEG-3 cells expressing LacZ, A12FL or A12Mut to specific human ECM proteins. A fluorimetric ECM protein binding assay utilizing a 96-well microtiter plate coated with collagen I, collagen II, collagen IV, fibronectin, laminin, tenascin and vitronectin was used to analyze cell-ECM binding. Pre-coated BSA served as a negative control. (B) Histograms showing α/β integrin binding affinities of EVTs and LacZ-, A12FL- and A12Mut-expressing JEG-3 cells. Cell-integrin adhesion was determined in 96-well plates coated with monoclonal antibodies specific to α (α1, α2, α3, α4, α5, αv, αvβ3) or β (β1, β2, β3, β4, β6, αvβ5, α5β1) integrins. ECM and integrin adhesion was quantified by lysing bound cells, incubating with the nucleic acid binding CyQuant GR dye, and reading fluorescence at 485/530 nm. Dotted line provides reference to baseline measurements (n = 3; * = P≤0.05, where * denotes a significantly different value compared to LacZ control values).

Different integrin heterodimer combinations allow for cells to acquire distinct ECM substrate preferences, and studies have shown in both cancer and trophoblast models that specific integrins regulate cell invasion [34]. Using fluorimetric 96-well microplate arrays pre-coated with specific ECM proteins (collagen I, collagen II, collagen IV, fibronectin, laminin, tenascin, and vitronectin) and monoclonal antibodies to integrin subtypes (α1, α2, α3, α4, α5, αv, αvβ3, β1, β2, β3, β4, β6, αvβ5, α5β1) we sought to determine differences in cell-binding affinities to these defined substrates elicited by ADAMTS-12. Wildtype EVTs isolated from first trimester chorionic villous explants served as a biological control for ECM binding affinities associated with an invasive phenotype. EVTs were shown to bind significantly to all the ECM proteins analyzed (Figure 6A), however, the binding affinities to collagen II, fibronectin and vitronectin were strongest. LacZ expressing JEG-3 cells bound to collagen I, collagen II, fibronectin, laminin and tensacin, but bound with low affinities to collagen IV and vitronectin (Figure 6A). Similar to EVTs, A12FL and A12Mut expressing JEG-3 cells bound significantly to all the ECM proteins. Notably, their binding affinity to collagen II, collagen IV and vitronectin were significantly greater than LacZ expressing cells (Figure 6A). Integrin cell adhesion was also shown to be different between ADAMTS-12 (A12FL and A12Mut) and LacZ expressing JEG-3 cells. Whereas EVTs bound with great affinity to all α integrin subunits (Figure 6B), LacZ-expressing JEG-3 cells failed to bind to α1, α4, and αv antibodies (Figure 6B). Upon exogenous expression of A12FL or A12Mut, a modest increase in binding to α3 was observed while binding to the αv antibody was greatly increased as was binding to αvβ3 (Figure 6B). Additionally, decreases in binding to the β2 antibody and to αvβ5 and α5β1 antibodies were observed. Taken together, these data demonstrate that ADAMTS-12, regardless of a functional catalytic domain, regulates specific cell-ECM binding differences resembling those of an invasive phenotype.

### αv integrin expression is controlled by ADAMTS-12

**Figure 7:**
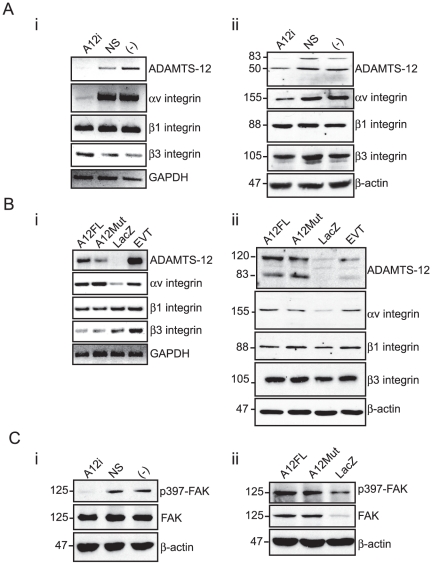
ADAMTS-12 regulates αv integrin expression and FAK activity. (A) Representative ethidium bromide stained agarose gels containing RT-PCR products (i) and Western blot autoradiographs (ii) respectively depicting mRNA and protein levels of ADAMTS-12, αv-, β1-, and β3-integrin subunits in EVTs untreated (-) or transfected with an siRNA oligonucleotide directed against ADAMTS-12 (A12i) or control non-silencing siRNA (NS). GAPDH and b-actin serve as endogenous mRNA and protein controls respectively. (B) Ethidium bromide stained gels and Western blot autoradiographs depicting mRNA (i) and protein levels (ii) of the genes described above in JEG-3 cells stably transfected with pcDNA3.1 constructs expressing LacZ, A12FL or A12Mut. EVTs served as an ADAMTS-12 positive control. (C) Representative autoradiographs of Western blots depicting phospho-397 FAK (p397) and total FAK (FAK) protein levels in the EVTs (i) and JEG-3 cells (ii) described above. The molecular weight markers (kDa) are shown to the left.

Since studies have shown that the av integrin subunit, and in particular, the αvβ3 integrin heterodimer plays an important role in EVT invasion [34], [35] and in light of our findings showing a significant increase in αvβ3 mediated cell adhesion (Figure 6), we investigated whether ADAMTS-12 regulates the expression of these integrin subunits. In EVTs where endogenous ADAMTS-12 was knocked down using siRNA, av integrin mRNA and protein levels showed a correlative decrease in expression compared to controls (Figure 7A). β1 and β3 integrin subunit levels were unaffected by a reduction in ADAMTS-12 expression (Figure 7A). Gain of ADAMTS-12 function in JEG-3 cells (A12FL and A12Mut) lead to an increase in mRNA and protein expression of the αv subunit, while β1 and β3 integrin subunit protein levels remained unchanged, although β3 integrin subunit mRNA levels in A12FL and A12Mut cells showed a modest decrease in expression (Figure 7B). Since phosphorylation of FAK (phospho-397) is an indicator of activated integrin signaling [33], we performed Western blot analysis on protein lysates harvested from EVTs transfected with ADAMTS-12 siRNA or JEG-3 cells expressing A12FL or A12Mut and assayed for phospho-397 FAK and total FAK expression (Figure 7C). Loss-of ADAMTS-12 in EVTs showed a marked decrease in FAK phosphorylation whereas overexpression of ADAMTS-12 lead to an increase in FAK phosphorylation. Although total FAK levels in EVTs remained unchanged, we did observe a proportional increase in total FAK compared to phosphorylated FAK in JEG-3 cells overexpressing A12FL and A12Mut. To investigate whether a physical interaction between ADAMTS-12 and αvβ3 integrin occurs, we imunoprecipitated αv integrin from protein lysates obtained from A12FL-expressing JEG-3 cells and probed for ADAMTS-12. IP analysis suggested that no physical interaction between ADAMTS-12 and αvβ3 occurs (Figure S3). Verification of αv pull-down was achieved by re-probing the immunoblot with a monoclonal αv-integrin antibody.

### ADAMTS-12 controls cell invasion through an αvβ3 integrin mediated mechanism

**Figure 8:**
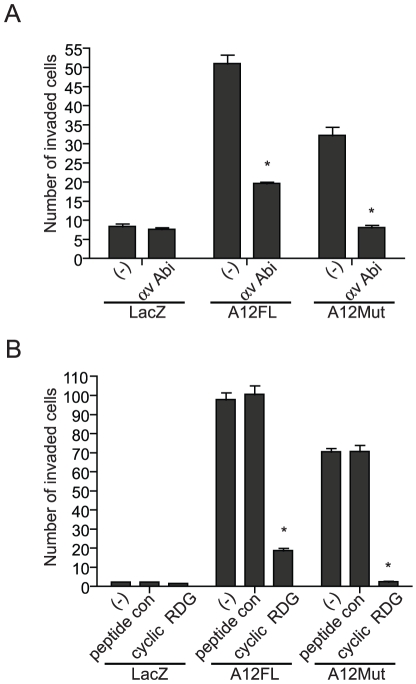
ADAMTS-12 promotes cell invasion through an αvβ3 integrin mediated mechanism. (A) A histogram representing the number of JEG-3 cells stably transfected with LacZ, A12FL or A12Mut that invaded through Matrigel-coated Transwell invasion chambers. Cells were cultured in complete DMEM media alone (-) or containing a function-perturbing antibody against the αv integrin subunit (αv Abi; 1∶50 dilution) for 48 h. (B) A histogram showing the invasiveness of the aforementioned JEG-3 cells in Matrigel-coated Transwell invasion chambers. Cells were cultured in complete DMEM media containing 5 nM of an αvβ3 integrin-specific bi-cyclic RGD peptide (cyclic RGD), control peptide (peptide con) or media alone (-) for 48 h. All assays were performed in triplicate and performed on three independent occasions (n = 3; * = P≤0.05, compared to controls).

Seeing that ADAMTS-12 regulates the expression and function of αvβ3 integrin, and that αvβ3 has been previously shown to promote an invasive phenotype in human EVTs, we next tested whether ADAMTS-12-mediated cell invasion is facilitated by αvβ3. Treatment of LacZ, A12FL or A12Mut expressing JEG-3 cells with a function-perturbing αv integrin antibody cultured in Matrigel-coated Transwells significantly inhibited A12FL- and A12Mut-mediated invasion, but had no affect on invasion of LacZ expressing cells (Figure 8A). In concordance with our previous data, A12FL and A12Mut showed a significant increase in cell invasion compared to LacZ, with A12FL demonstrating a slightly more invasive phenotype over A12Mut. The effect on cell invasion of a bi-cyclic RGD peptide that demonstrates preference towards the αvβ3 integrin heterodimer had a similar affect to that of the αv perturbing antibody (Figure 8B). Specifically, peptide treatment did not affect LacZ cell invasion, but significantly inhibited A12FL and A12Mut invasion. Interestingly, the number of αvβ3 peptide-treated cells that invaded through the Matrigel-coated insert were higher in the A12FL population than in the A12Mut population, suggesting that a component of ADAMTS-12-mediated invasion is dependent on its catalytic domain. Any advantage that A12Mut provided in JEG-3 cell invasion was completely abolished with bi-cyclic RGD peptide treatment.

## Discussion

Multiple ADAMTS (ADAMTS-1, -2, -4, -5, -6, -7, -9, and -12) were detected in tissue samples of first trimester human placenta. With the exception of ADAMTS-5, this repertoire of ADAMTS subtypes was maintained, albeit at differing levels, in cultures of highly invasive EVTs propagated from these tissues and in poorly invasive JEG-3 cells. Although the overall biological significance of this expression pattern of ADAMTS proteins in the human placenta remains to be elucidated, our studies demonstrate that ADAMTS-12 plays a non-redundant role in human trophoblastic cell invasion *in vitro*. In addition, ADAMTS-12 mRNA levels in EVTs are differentially regulated by TGF-β1 and IL-1β in a concentration- and time-dependent manner. Thus, ADAMTS-12 is tightly regulated in human trophoblastic cells and signaling pathways that control invasion act, at least in part, by downregulating ADAMTS-12 gene expression.

In agreement with our findings, ADAMTS-2 mRNA has been detected in first trimester placenta [36], with mRNA transcripts encoding ADAMTS-1, -4, -5, -6, -7, -9, and -10 mRNA also being found in human term placenta [37]–[41]. In addition to these ADAMTS subtypes, we found that ADAMTS-12 is present in placenta obtained during the first trimester of pregnancy and in primary cultures of EVTs propagated from these tissues. Cal *et al* [21] failed to detect ADAMTS-12 mRNA transcripts in normal human tissues that included term placenta, but found it exclusively in fetal lung. Additionally, ADAMTS-12 expression has been detected in normal cartilage where it is proposed to regulate chondrocyte differentiation [42]. Differences in placental ADAMTS-12 mRNA levels are likely attributable to the changes in the subpopulations of cytotrophoblasts that constitute this dynamic tissue throughout gestation. In particular, EVTs predominate during the first trimester with the number of these cells declining sharply thereafter and being absent in term placental tissues [1]. Failure to detect ADAMTS-5 in human trophoblastic cells *in vitro* suggests that the expression of this ADAMTS subtype in the placenta is restricted to one or more of the other cellular compartments that comprise this dynamic tissue, particularly the mesenchymal core and/or vasculature. Molecular weight discrepancies between endogenous ADAMTS-12 protein species in EVTs and first trimester placenta (35 kDa major product) compared to exogenous ADAMTS-12 in JEG-3 cells (83 kDa major product) were observed. This could in part be explained by the different cell types analyzed and variability in expression of proteolytic processing enzymes. Artificially high levels of over-expressed ADAMTS-12 may saturate proteolytic processes resulting in a decrease in these cleavage products.

In contrast to its restricted expression in normal human tissues, ADAMTS-12 mRNA is readily detectable in the cartilage and synovium of patients with arthritis and in cancer cell lines of diverse origin with levels of this mRNA transcript being present at higher levels in gastric carcinomas compared to matched normal tissue controls [21]. These later findings suggest a putative role(s) for ADAMTS-12 in the development of an invasive cellular phenotype, and indeed our studies revealed that manipulating the function of this ADAMTS subtype ascribed a pro-invasive phenotype in human trophoblastic cells. Altered expression levels of other ADAMTS subtypes have also been detected in human carcinomas but their individual contribution(s) to the onset and progression of cancer remains unclear [7], [9], [10], [43]–[45]. For example, ADAMTS-1 mRNA levels have been shown to be either increased [41] or decreased [9] in breast carcinomas. Higher levels of this ADAMTS subtype have also been associated with pancreatic and hepatocellular cancer [43] whereas ADAMTS-1 mRNA levels are unchanged in the onset and progression of kidney cancer [46] and decreased in lung carcinomas [41]. Exogenous expression of ADAMTS-1 has been shown to decrease the experimental metastasis of Chinese hamster ovary cells [46] but increase the metastatic potential of mammary and lung cancer cell lines *in vivo* [47] whereas our study demonstrates that increased expression levels of ADAMTS-1 do not alter the invasive capacity of human trophoblastic cells *in vitro*. A possible explanation for these contradicting observations involves auto-proteolytic processing that can account for pro- or anti-metastatic effects depending on the cleavage site [46]. It is proposed that proteolytic cleavage of the substrate binding site in ADAMTS-1 impairs substrate binding and unveils anti-angiogenic TSP-1 motifs.

Recent studies characterizing the role of ADAMTS-12 in tumor progression demonstrated an anti-tumorigenic role for this subtype [48]. Llamazares *et al* showed ADAMTS-12 to inhibit HGF-mediated scattering of MDCK cells and also prevented bovine aortic endothelial cells from forming tubules in response to VEGF. Additionally, subcutaneous injection of SCID mice with the highly aggressive lung carcinoma A549 cell line over-expressing ADAMTS-12 repressed tumor growth. More recently, El Hour *et al* [49] characterized the role of ADAMTS-12 in regulating tumorigenesis using a malignant keratinocyte model system in ADAMTS-12 null mice. Although this study did not specifically address cell invasion, it did shed light on the importance of how ADAMTS-12 in the tumor microenvironment inhibits key aspects of tumorigenesis, such as cell proliferation and angiogenesis. In lieu of novel data describing ADAMTS-12 as a putative tumor suppressor gene expressed predominantly in the stroma [49], [50], further studies are required to evaluate the biological and clinical significance of (dys)regulated expression levels of ADAMTS-12 in the onset and/or progression of cancer to the later stages of the disease state, and to delineate the function of this ADAMTS subtype in processes such as cell migration, invasion, and growth in other *in vivo* tumor model systems.

Our demonstration that ADAMTS-12 modulates both cell-ECM binding and cell invasion through an RGD-dependent mechanism is an intriguing finding as neither the disintegrin-like domain, the TSP-repeat domains nor any other domain of any characterized ADAMTS metalloproteinase contain RGD-like motifs [8]. It is therefore unlikely that ADAMTS-12 promotes an invasive phenotype directly through an RGD-dependent interaction, but rather regulates the function or expression of proteins capable of controlling RGD-mediated cell-matrix adhesion and cell invasion. Our work demonstrated that ADAMTS-12 regulates cell-ECM binding to specific ECM substrates and cell attachment facilitated by specific integrin cell adhesion molecules. Specifically, we showed that ADAMTS-12 expressing trophoblastic cells bind more readily to collagen II, collagen IV and vitronectin and up-regulate the expression of αv and αvβ3 integrins on their cell surface. Furthermore, this up-regulation is associated with an increase in integrin signaling activity and cell invasion. Collagen IV is a basement membrane protein whereas collagen II is a fibrillar collagen predominantly found in cartilage connective tissue and interacts predominantly with β1 or β2 integrins [51], [52]. While we did not observe any changes in β1 or β2 integrin-mediated binding in ADAMTS-12 transfected JEG-3 cells, this conflicting finding may be explained by the ability of EVTs to synthesize the collagen II degrading protease, MMP-13 [53]. Collagen II remodeling by MMP-13 may result in the uncovering of cryptic integrin-binding sites that may lead to increased cell-matrix adhesion by integrins other than β1 or β2. Collagen interactions mediated by other integrins absent in our microplate integrin adhesion assay may provide additional explanations as to why we observed increases in cell binding to collagens II and IV. For example, α10β1 and α11β1 integrins have been shown to bind to these collagens *in vitro* [54]. Lastly, the association of ADAMTS-12 with the ECM might lead to proteolytic-independent conformational ECM changes that may also result in uncovering cryptic integrin binding sites.

Vitronectin is an RGD-containing multifunctional protein found in serum and extracellular matrices, and is secreted by trophoblastic cells during the first trimester of pregnancy [55], [56]. It has been demonstrated that integrin receptors containing RGD binding motifs (ie. αvβ3, αvβ5, α5β1 and αIIbβ3) bind to vitronectin [52]. The αvβ3 integrin has been shown to play important roles in promoting both trophoblast and cancer cell invasion [34], [57]–[61]. Binding of αvβ3 integrin to its ligands results in the activation of mitogen-activated protein kinase (MAPK) and focal adhesion kinase (FAK) [56]. Kabir-Salmani *et al* [35] demonstrated that IGF-I-mediated migration of trophoblasts is αvβ3 integrin-dependent and is associated with αvβ3 integrin heterodimer co-localization with phosphorylated FAK, paxillin and viniculin at focal adhesions. These data, along with the findings in our study, provide strong evidence in assigning a key role for the αvβ3 integrin in promoting an invasive phenotype in human trophoblasts. Activation of FAK is facilitated by many β1 and β3-containing integrins and is therefore not a singular αvβ3 event [62]. Analysis of FAK activation in our cell culture system should be interpreted only as an indicator for the acquisition of an invasive phenotype facilitated by ADAMTS-12. In light of these observations, αvβ3 integrin appears to play key roles in facilitating cell invasion processes in both physiological and pathological contexts. Recent findings that have shown ADAMTS proteinases to regulate cell motility through Ras-MAPK signaling events by mechanisms independent of their MMP-like domain support our findings [63]. Exactly how ADAMTS-12 regulates integrin expression remains to be elucidated. Our data would suggest that ADAMTS-12 acts in a cell automomous manner regulating ECM activity independent of its endogenous proteolytic function, however more studies are required to fully interrogate the mechanisms by which ADAMTS-12 regulates αv integrin transcription and function.

In summary, we have determined that ADAMTS-12, independent of its proteolytic activity, plays a critical role in human trophoblastic cell invasion *in vitro*. This study not only provides an insight into the molecular mechanism underlying epithelial cell invasion but also extends our understanding of the cell biology and complexity of the ADAMTS gene family. Furthermore, the trophoblastic cell cultures examined in these studies offer an ideal model to further dissect the contributions of the distinct domains of ADAMTS-12 in regulating epithelial cell invasion.

## Supporting Information

Figure S1 **Exogenous ADAMTS-12 does not alter cellular aggregation.** Photomicrographs show JEG-3 cells stably transfected with pcDNA3-ADAM-TS12 (A12FL), pcDNA3-ADAM-TS12-MUT (A12Mut) or pcDNA3-LacZ (LacZ) that were cultured in 20 µl hanging drops for 2 or 8 h prior to being inverted and mounted with a glass cover slip. The histogram quantitatively describes cellular aggregation assayed at 2 h as determined by grouping cell aggregates into three categories (<5 cells/aggregate, 5–15 cells/aggregate, >15 cells/aggregate). Hanging drop assays were done in triplicate and the experiment was performed on three independent occasions (n = 3).
(TIFF)

Figure S2 **ADAMTS-12 does not regulate the expression of a subset of ECM proteins.** Ethidium bromide-stained photographs of RT-PCR products generated using primers specific to the ECM proteins vitronectin (V), fibronectin (F), laminin (L) and tenascin (T) and the housekeeping gene GAPDH from cDNA's made from (A) JEG-3 cells stably transfected with ADAMTS-12 (A12FL) or control LacZ or from (B) cDNA's made from EVTs or JEG-3 cells. GAPDH levels (C) provide PCR product standardization between lanes. *L*; DNA ladder. PCR reactions were performed on two separate occasions (N = 2).
(EPS)

Figure S3 **ADAMTS-12 does not associate with the αv integrin.** αv integrin was immunoprecipitated from protein lysates of JEG-3 cells stably transfected with LacZ (lanes 1 and 2), A12FL (lanes 3 and 4) or A12Mut (lanes 5 and 6). Immunoprecipitates (IP) were probed for with antibodies directed against ADAMTS-12 (A) and αv integrin (B). Representative autoradiographs from three independent experiments (N = 3) as described for *A* and *B* are shown. The molecular weight markers (kDa) are shown to the left. *IB*, immunoblot.
(EPS)

Table S1 **Names of genes, DNA primer sequences and PCR conditions for the semiquantitative analysis of levels of mRNA transcripts for ADAMTS, integrin and ECM genes in human placental tissue and trophoblastic cells.**
(DOC)
