# Supplementary figures and images for: Regulated Expression of ADAMTS-12 in Human Trophoblastic Cells: A Role for ADAMTS-12 in Epithelial Cell Invasion?
Source: PLoS One. 2011 Apr 11;6(4):e18473. doi: 10.1371/journal.pone.0018473 (PMC3073978; doi:10.1371/journal.pone.0018473)

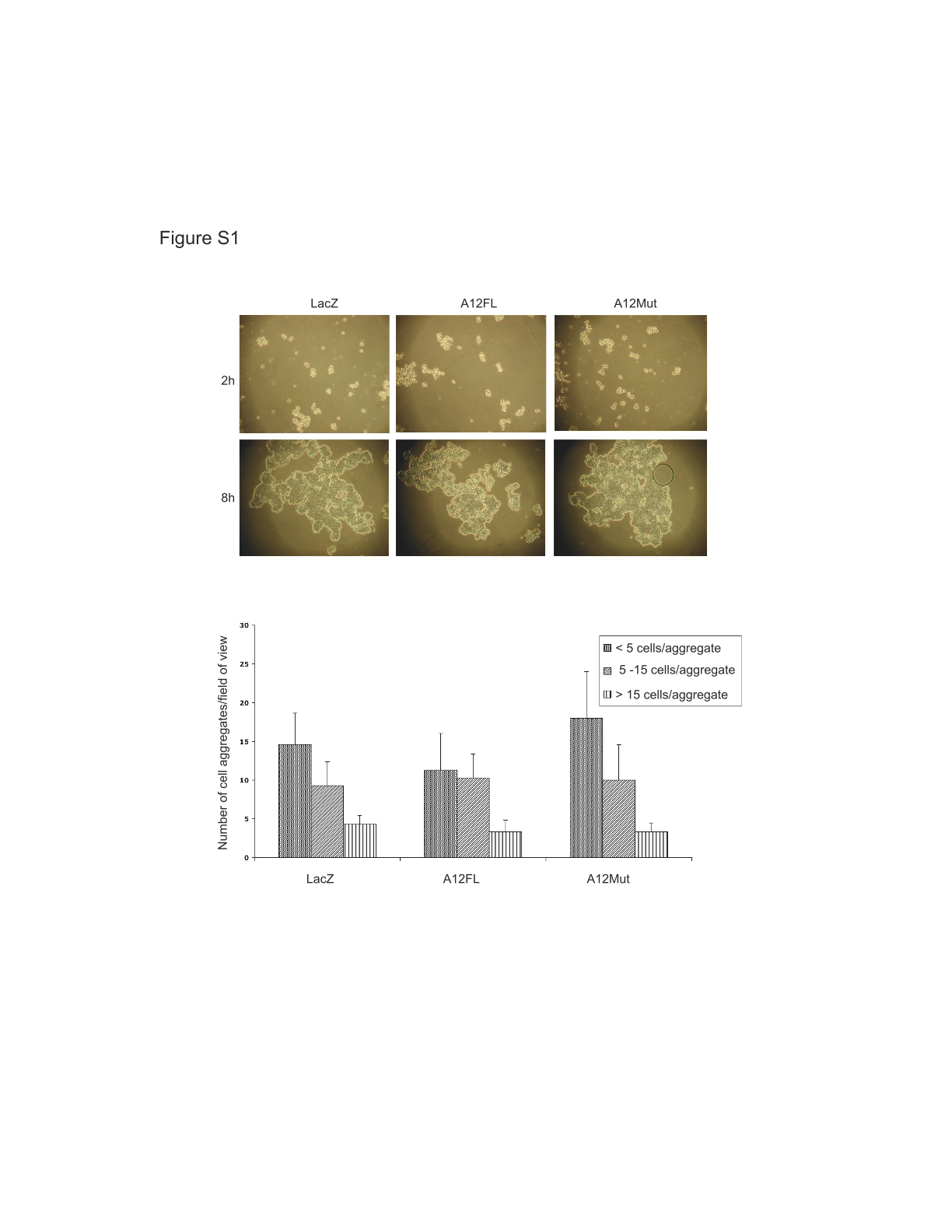

Supplement: Figure S1 — Exogenous ADAMTS-12 does not alter cellular aggregation. Photomicrographs show JEG-3 cells stably transfected with pcDNA3-ADAM-TS12 (A12FL), pcDNA3-ADAM-TS12-MUT (A12Mut) or pcDNA3-LacZ (LacZ) that were cultured in 20 µl hanging drops for 2 or 8 h prior to being inverted and mounted with a glass cover slip. The histogram quantitatively describes cellular aggregation assayed at 2 h as determined by grouping cell aggregates into three categories (<5 cells/aggregate, 5–15 cells/aggregate, >15 cells/aggregate). Hanging drop assays were done in triplicate and the experiment was performed on three independent occasions (n = 3). (TIFF) [file pone.0018473.s001.tif]
