# Supplementary material for: Regulated Expression of ADAMTS-12 in Human Trophoblastic Cells: A Role for ADAMTS-12 in Epithelial Cell Invasion?
Source: PLoS One. 2011 Apr 11;6(4):e18473. doi: 10.1371/journal.pone.0018473 (PMC3073978; doi:10.1371/journal.pone.0018473)
Supplement: Table S1 — Names of genes, DNA primer sequences and PCR conditions for the semiquantitative analysis of levels of mRNA transcripts for ADAMTS, integrin and ECM genes in human placental tissue and trophoblastic cells. (DOC) [file pone.0018473.s004.doc]

**Table S1.**

| Gene | Primer Sequence | Estimated PCR product size (bp) | PCR conditions |
| --- | --- | --- | --- |
| ADAMTS-1 | Forward: 5’-CGAGTGTGCAAAGGAAGTGA-3’  Reverse: 5’-CTACCCCATAATCCCACCT-3’ | 399 | Denaturing: 94°C 30s  Annealing: 64°C 30s  Extension: 72°C 60s  28 cycles |
| ADAMTS-2 | Forward: 5’-CCTATGACTGGCTGCTGGAT-3’  Reverse: 5’-CTCCCAAAGTGCTGGGATAA-3’ | 310 | Denaturing: 94°C 30s  Annealing: 62°C 30s  Extension: 72°C 60s  30 cycles |
| ADAMTS-4 | Forward: 5’-AATCCAGGGTGGTGGTGATA-3’  Reverse: 5’-TACTCAGGAGGCTGAGGCAT-3’ | 349 | Denaturing: 94°C 30s  Annealing: 60°C 30s  Extension: 72°C 60s  30 cycles |
| ADAMTS-5 | Forward: 5’-GCCCATGGTAACTGTTTGCT-3’  Reverse: 5’-CCTCTTCCCTGTGCAGTAGC-3’ | 444 | Denaturing: 94°C 30s  Annealing: 64°C 30s  Extension: 72°C 60s  35 cycles |
| ADAMTS-6 | Forward: 5’-TGACAGTCCAGCACCTTCAG-3’  Reverse: 5’-GCAGGAGCACGTTCAGTGTA-3’ | 249 | Denaturing: 94°C 30s  Annealing: 55°C 30s  Extension: 72°C 60s  30 cycles |
| ADAMTS-7 | Forward: 5’-CCATGTGGTGTACAAGCGTC-3’  Reverse: 5’-GGTCCTTCCTCCTCATCTTCC-3’ | 389 | Denaturing: 94°C 30s  Annealing: 58°C 30s  Extension: 72°C 60s  35 cycles |
| ADAMTS-9 | Forward:5’-ACCCGGATGATGAGATACGT-3’  Reverse: 5’-CCACAGGTCACAGAGCAAGA-3’ | 161 | Denaturing: 94°C 30s  Annealing: 62°C 30s  Extension: 72°C 60s  28 cycles |
| ADAMTS-12 | Forward: 5’-GTGCAGCGAGGAGTACATCA-3’  Reverse: 5’-GCGTTTTCTTTCTCCAGTGC-3’ | 488 | Denaturing: 94°C 30s  Annealing: 63°C 30s  Extension: 72°C 60s  28 cycles |
| αv integrin | Forward:5’-AGATGTTGGGCCAGTTGTTC-3’  Reverse:5’-GCAACTCCACAACCCAAAGT-3’ | 321 | Denaturing: 94°C 45s  Annealing: 62°C 45s  Extension: 72°C 90s |
| β1 integrin | Forward:5’-TTGGGTACACGATGCAGGTA-3’  Reverse:5’-CTGGTTGATGTCACCAATGC-3’ | 420 | Denaturing: 94°C 45s  Annealing: 60°C 45s  Extension: 72°C 90s |
| β3 integrin | Forward:5’-GGCCATGTGACCTGAACTTT-3’  Reverse:5’-CATCTCCCACCCTAGTCCAA-3’ | 397 | Denaturing: 94°C 45s  Annealing: 55°C 45s  Extension: 72°C 90s |
| Vitronectin | Forward: 5’-AACACTTTGCCATGATGCAG-3’  Reverse: 5’-ACTCTGGATGGGTTCACAGG-3’ | 398 | Denaturing: 94°C 45s  Annealing: 55°C 45s  Extension: 72°C 90s  28 cycles |
| Laminin | Forward: 5’-TGGCGAGCCTACTAAAGGAA-3’  Reverse: 5’-TTCTTCGCATCAACTGCATC-3’ | 363 | Denaturing: 94°C 45s  Annealing: 58°C 45s  Extension: 72°C 90s  28 cycles |
| Tenascin | Forward: 5’-TCTCAGGGTCATTCACCACA-3’  Reverse: 5’-AGGTAACCGGTGACTGATGC-3’ | 382 | Denaturing: 94°C 45s  Annealing: 60°C 45s  Extension: 72°C 90s  35 cycles |
| Fibronectin | Forward: 5’-TCGAGGAGGAAATTCCAATG-3’  Reverse: 5’-CTCTTCATGACGCTTGTGGA-3’ | 382 | Denaturing: 94°C 45s  Annealing: 58°C 45s  Extension: 72°C 90s  28 cycles |
| GAPDH | Forward: 5’-CCCAATTCTCTACGGAGTCG-3’  Reverse: 5’-AATCTCCCAGGGTTGCTTCT-3’ | 378 | Denaturing: 94°C 45s  Annealing: 55°C 30s  Extension: 72°C 60s  20 cycles |
